# Medical Emergencies in Goa

**DOI:** 10.4103/0970-0218.62555

**Published:** 2010-01

**Authors:** Sahoo Saddichha, Mukul Kumar Saxena

**Affiliations:** Division of Clinical Research, Emergency Management and Research Institute, Hyderabad, Andhra Pradesh, India

**Keywords:** Emergencies, Goa, medical emergency

## Abstract

**Background:**

Most emergencies in Goa arise due to road traffic accidents and drowning, which have been compounded by the rise in number of recorded accidents in 2007 to be above 4000. It is believed that 11 people meet with an accident on Goa's roads every day and this is expected to rise by 10% by next year. Similar is the case with drownings and other medical emergencies. We therefore aimed to conduct a cross-sectional survey of medical emergencies and identify various types of emergencies presenting to emergency departments.

**Materials and Methods:**

Using a stratified random sampling design, all emergencies presenting to the three government hospitals in Goa, which handle 90% of all emergencies currently, were studied on specially designed data sheets in order to collect data. Emergency medical technicians (ETs) were placed in the Casualty Ward of the medical colleges and they recorded all emergencies on the data sheet. The collected data were then analyzed for stratification and mapping of emergencies.

**Results:**

GMC Hospital attended to majority of emergencies (62%), which were mainly of the nature of accidents or assaults (17%) and fever related (17%). Most emergencies were noncritical and about 1% expired. Maximum emergencies also presented from Salcette and Bardez, and occurred among young males in the age group of 19-45 years. Males were also more prone to accidents while females had pregnancies as emergencies.

**Conclusion:**

Potential emergency services need to target young males with higher concentrations required in Salcette in South Goa and Bardez in North Goa.

## Introduction

Goa, India's smallest but richest per capita state with a population of 1.3 million, became a part of India only in 1961, before which it was governed directly from Portugal. The state of Goa, influenced by the colonial Portuguese rule for over 450 years, consumes a large quantity of alcohol and has also accepted alcohol as a social beverage, the repercussions of which are obvious today. One of the most serious of these has been the massive increase in the number of road traffic accidents and accidental drowning, which commonly present as medical emergencies to hospitals.

Most common emergencies that occur in any state have been reported to be pregnancies (22.7%), accidents (12.2%), or assaults (15.4%) and fever related.(1) Two small studies from Chennai indicated that fever and gastroenteritis(2) and trauma and cardiovascular etiologies(3) were the most common emergencies presenting to the emergency department. There are scant data regarding the epidemiology from other states including Goa. However, indirect data from Goa indicate that both road traffic accidents and drowning are most commonly noted.(4–7)

A rapid explosion of road traffic accidents has been observed with accidents doubling from 2800 in 2001 to above 4000 in 2007.(4) In the year 2007, there have been about 4000 accidents, an 8% rise over last year with more than 320 persons killed, a rise of over 6% over the previous year.(5) It is believed that 11 people meet with an accident on Goa's roads every day and this is expected to rise by 10% by next year. The Police Department has reported that till date in 2008, there have been approximately 150 fatal accidents.(6) Most accidents involve mainly two-wheelers in Goa and occur around 8 pm in the night. In addition, there have been at least one or two drowning deaths per week accounting for a large number of accidental deaths in Goa. The Director, Goa tourism department, has reportedly informed the media that most drownings are fatal due to lack of medical attention at the right time.(7) A large number of both forms of accidental deaths have been linked to the wide epidemic of substance use, which in turn increases both mortality and morbidity rates. The outcome also depends to a large extent on the mode of transport and delay in getting to a hospital.

Emergency Management and Research Institute (EMRI) has been providing comprehensive emergency services, in partnership with various state governments, by running a single toll-free number 108. Since 2005, EMRI has started services in nine states of India and has initiated services in Goa since September 2008. Prior to launch, a comprehensive study of emergencies was required in order to map out strategies and placement of ambulances. Since Goa has an area of just 3702 sq. km serving a population of 1.3 million,(8) a study of this nature can tap most emergencies in the state.

This study therefore attempted to evaluate current emergencies presenting to government emergency departments to

1. identify various types of medical emergencies occurring in Goa,
2. identify specific variables associated with these emergencies in order to put in place additional measures to reduce number of deaths,
3. identify and target specific causes for intervention, and
4. explore differences in the patterns of presentations to various hospitals in Goa.

## Materials and Methods

Using a stratified random sampling design, records of all medical emergencies presenting to the three government hospitals, namely, Goa Medical College Hospital (GMCH), Asilo Mapusa, and Hospicio Margoa, were prospectively studied on specially designed data sheets in order to collect data. These hospitals currently serve as a primary referral centre for nearly 90% of all reported emergencies. Trained paramedic personnel or emergency medical technicians (EMTs) were utilized for the purpose of data collection after a detailed training on using the data sheet. Once trained, these EMTs were then placed in the Casualty Ward of the three government hospitals in three continuous 8-h shifts. Data were collected over a 4-day period from September 01-04, 2008, with a 24-h round-the-clock collection ensuring that no cases went missing. All data so collected by the end of the study period were entered into a database and further analyzed to detail type of emergency, location of emergency, and other variables associated with medical emergencies.

## Results

A total number of 360 emergencies were seen over a 60-h period. A majority of the emergencies were handled by GMCH (62%) followed by Asilo Mapusa (23%) and Hospicio Margoa (15%). A higher prevalence of emergencies was also noted among males (64%) and in the 19-30 and 31-45 year age groups which accounted for nearly 60% of all emergencies [Table 1].

**Table 1:** Sociodemographics and clinical analysis of emergencies

Sociodemographic and clinical variables	Prevalence (%)
Gender distribution	
Males	64.5
Females	35.5
Age distribution (years)	
<10	12
11-18	07
19-30	38
31-45	21
46-60	15
>60	07
Types of emergencies	
Abdominal pain	08
Accidents and assaults	17
Cardiovascular	09
Poisoning and overdose	01
Falls and fractures	10
Pregnancy related	07
Fevers	17
Animal bites	05
Head injuries	03
Others	23
Hospital distribution	
Goa Medical College Hospital	62.3
Asilo Mapusa	23.2
Margoa Hospital	14.5
Status at hospital	
Noncritical	90
Critical	09
Expired	01

When categorized into types of emergencies, most emergencies were either due to vehicular accidents and assaults (17%) or fever related (17%). The other types of emergencies presenting to hospitals were mainly cardiac-related causes, falls and fractures, pregnancies, and abdominal pain. After being brought to the hospital and on evaluation, most emergencies were found to be noncritical in nature (90%). However, about 1% arrived dead at the hospital [Table 1].

### Stratification of emergencies by talukas

A stratification of emergencies by talukas resulted in the maximum emergencies presenting from Salcette and Bardez, each accounting for above 20% of all emergencies. Tiswadi accounted for about 15-20% of emergencies, while a lower prevalence was seen in the remaining talukas [Table 2]. These numbers are also compared with the percentage of population seen in these areas.

**Table 2:** Taluka distribution of emergencies

Talukas	Percentage to total population (%)	Prevalence of emergencies (%)
Pernem	5.3	3.1
Tiswadi	11.9	16.8
Bardez	16.9	23.2
Bicholim	06.7	03.9
Satari	04.3	01.7
Ponda	11.1	07.3
Sanguem	04.7	03.9
Quepem	05.5	05.9
Salcette	19.4	24.3
Mormugao	10.7	06.1

**Figure 1:**
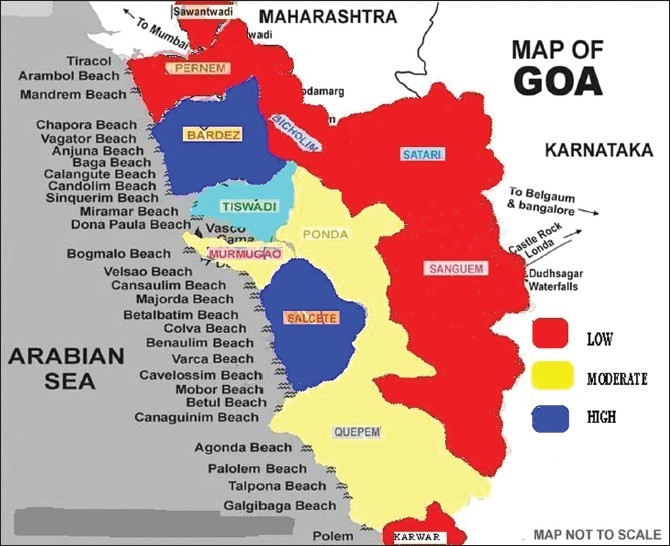
Mapping of emergencies

Based on the prevalences of emergencies, a mapping of the state was carried out [Figure 1], with red depicting “low prevalence”, blue “high” and yellow “moderate” prevalence.

### Emergency analysis

An in-depth analysis of each individual emergency was then carried out to study specifics of each kind of emergency. As has been mentioned before, the most common emergencies reported were accidents and assaults and cardiac-related emergencies. These were then compared for differences in gender, age, and area of occurrence [Table 3].

**Table 3:** Analysis of emergencies

Emergency details	Prevalence (%)
Accidents and assaults	
Gender distribution	
Males	79.3
Females	20.7
Age distribution (years)	
<10	12.5
11-18	03.6
19-30	33.9
31-45	23.2
46-60	19.6
>60	07.1
Taluka distribution	
Pernem	03.3
Bardez	09.8
Bicholim	01.6
Tiswadi	11.5
Ponda	06.6
Salcette	39.3
Quepem	14.8
Sanguem	01.6
Mormugao	03.3
Karwar	03.3
Sawantwadi	04.9
Cardiovascular emergencies	
Gender distribution	
Males	76
Females	24
Age distribution (years)	
<18	03.7
19-30	14.8
31-45	33.3
46-60	29.6
>60	18.5
Taluka distribution	
Mormugao	08.8
Bardez	26.5
Bicholim	05.9
Tiswadi	14.7
Ponda	02.9
Salcette	29.4
Quepem	08.8
Sanguem	02.9

### Accidents and assaults

Our study observed that accidents and assaults were more common in males (79%), in age groups of 19-45 years (57%) with a mean age of 33.1 + 17.3 years. GMCH was the main hospital attending to the majority of victims (71%), with 1 in every 7 accident victims arriving at the hospital in a critical condition. Accidents were also observed to be concentrated in the Salcette taluka (39%) with the rest distributed across the other talukas.

### Cardiovascular emergencies

This study observed that most cardiac emergencies occurred in males (76%), with a 3.1:1 male:female ratio. Further, cardiac emergencies were the highest in the age groups of 31-45 years (33%) and 46-60 years (30%), with a mean age of 46 (+16) years. Nineteen percent of all cardiac complaints were from the geriatric group of above 60 years. Most cardiac emergencies arose from Salcette (30%), Bardez (27%), and Tiswadi (15%). GMCH again was the main hospital serving cardiac emergencies (78%), with 1 in every 10 emergencies arriving in a critical condition.

### Gender differences in emergencies

**Figure 2:**
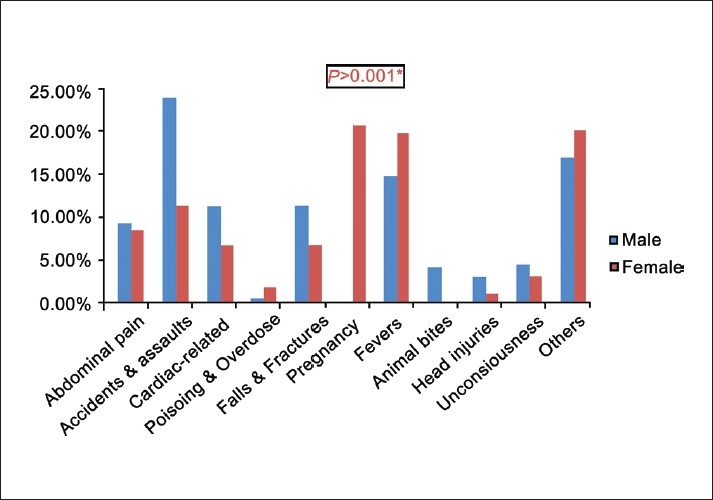
Gender differences in emergencies

We then analyzed different variables to study for any differences on types of emergencies. Based on gender, we observed a significant difference in the kinds of emergencies experienced. Whereas males had more emergencies related to accidents (24%), fevers (15%), and falls (11.5%), females had more emergencies related to both pregnancies (21%) and fevers (20%) [Figure 2].

### Age differences in emergencies

**Figure 3:**
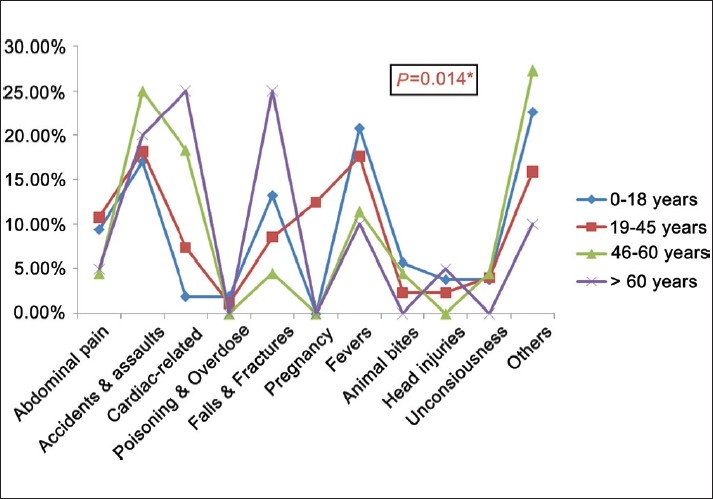
Age differences in emergencies

The most common cause of emergencies [Figure 3] in the young age group of below 18 years was reported to be fevers (20.8%) and accidents (17%), while for the adult age group of 19-45 years, it was observed to be accidents (18%) and fevers (17.5%). Similarly, in the middle age group of 46-60 years, the most common emergencies were accidents (25%) and cardiac related (18%), while in the geriatric age group, it was accidents and assaults (19%) and fevers (17%).

### Taluka differences in emergencies

A look at the various talukas from where the emergencies arose reveals interesting findings. Accidents were more common in Pernem, Salcette, Quepem, Karwar, and Sawantwadi while cardiovascular emergencies were more common in Mormugao. Pregnancy was more common in Sanguem while fevers were seen in Satari and Tiswadi [Table 4].

**Table 4:** Taluka differences in emergencies

	Abdominal pain (%)	Accidents/assaults (%)	Cardiac related (%)	Poisoning/overdose (%)	Falls and fractures (%)	Pregnancy (%)	Fevers (%)	Animal bites (%)	Head injuries (%)	Unconsciousness (%)	Others (%)
Pernem	9.1	18.2	0.0	9.1	0.0	9.1	0.0	9.1	9.1	18.2	18.2
Bardez	4.8	7.2	10.8	0.0	14.5	7.2	13.3	12.0	4.8	3.6	21.7
Bicholim	21.4	7.1	14.3	0.0	14.3	7.1	14.3	0.0	0.0	14.3	7.1
Satari	0.0	0.0	0.0	0.0	16.7	0.0	33.3	16.7	16.7	0.0	16.7
Tiswadi	11.7	11.7	8.3	0.0	10.0	10.0	25.0	1.7	0.0	5.0	16.7
Ponda	19.2	15.4	3.8	0.0	7.7	11.5	7.7	11.5	7.7	3.8	11.5
Salcette	8.0	27.6	11.5	0.0	6.9	4.6	18.4	0.0	2.3	5.7	14.9
Quepem	4.8	42.9	14.3	0.0	14.3	9.5	4.8	0.0	0.0	0.0	9.5
Sanguem	0.0	7.1	7.1	7.1	7.1	14.3	14.3	7.1	0.0	0.0	35.7
Mormugao	4.5	9.1	13.6	4.5	9.1	0.0	0.0	4.5	4.5	0.0	36.4
Karwar	16.7	33.3	0	0.0	0.0	0.0	0.0	0.0	0.0	0.0	0.0
Sawantwadi	0.0	37.5	0	0.0	12.5	0.0	0.0	0.0	0.0	0.0	25.0

## Discussion

Located in Panjim, Goa Medical College Hospital serves the whole of Goa and has possibly the maximum number of facilities and specialties available. It's no surprise therefore that GMCH attracts all kinds of emergencies and from all places in Goa including the border areas of Sawantwadi in Maharashtra and Karwar in Karnataka. However, there are certain questions that would need to be answered before definitive conclusions can be drawn such as

- Are victims coming to GMCH because of proximity of the medical college to the scene of emergency?
- Is it because there is perception in the community that the management at GMCH is better than other hospitals?
- Is it because the medical college is better equipped?

Since higher prevalence of emergencies were noted in the productive age groups, this calls for an urgent need to address prevention policies aimed at this vulnerable group of males aged between 19 and 45 years. Although fever appears as one of the top causes of emergency, better prehospital care and intervention by emergency medical services may reduce the attendance at emergency departments, thereby increasing the attention to more serious emergencies such as accidents and cardiovascular emergencies. Having observed a current expired rate of 1% and a critical status of 9%, awareness needs to be generated on the usefulness of prehospital care on prognosis and outcome of emergencies, which would definitely help in saving more lives.

The mapping of the state revealed that low-prevalence emergencies (in red) were not only concentrated along the border [Figure 1], but also located farther away from the location of the main hospitals in Goa. On the other hand, the maximum emergencies were reported from the same districts in which the main hospitals were located (in blue). It was also observed that there is a distinct middle zone (in yellow) separating the high- from the low-prevalence zones. The distance and lack of organized ambulance services in the peripheral areas may perhaps explain the low prevalence in the border districts while the opposite may be true for the high-prevalence ones. Since Salcette and Bardez also accounted for the highest emergencies, a positioning of paramedic personnel and ambulances with an easy access to these areas would go a long way in saving potential lives. However, one can conclude that most areas had similar prevalence of emergencies as compared to the ratio of their population to the total population of Goa.

Among emergencies, accidents and assaults were more common in young males, which incidentally is the most productive age group and can result in a serious loss of breadwinners to the family. It has been observed that males experience maximum interpersonal violence.(9) Such experiences result in an increased risk of being involved in accidents or assaults. Programs designed to educate on means of coping with stress may reduce some occurrence of interpersonal violence. We also observed most accidents occurring around major tourist spots and highways, putting the onus on the traffic managers and public health specialists to devote attention to this area in order to reduce these numbers.

Similarly, cardiovascular emergencies were also most common among older adults. The mean age of cardiovascular emergencies is not surprising (46 years), considering that Indians often present with cardiovascular emergencies at a younger age than the rest of the world.(10–13) However, this would call for renewed efforts to target cardiac care at the vulnerable population and promote better life styles and eating habits.

## Conclusion

This study attempted to perform a rapid cross-sectional situational analysis of factors contributing to medical emergencies in Goa. Using a sampling design spread over a duration of 4 days and covering about 90% of all emergencies in Goa, this study attempts to give a current view of medical emergencies in the state. Since the casualties record all emergencies presenting to the hospitals, a round-the-clock collection of data ensured that all emergencies were accounted for. Further, the presence of trained paramedic personnel ensured that emergencies were handled with sensitivity and accurate clinical data were collected.

This study demonstrates the urgent need for prehospital emergency services which should target young males, since this population is especially vulnerable to different types of emergencies. A higher concentration is also required in Salcette in South Goa and Bardez in North Goa with the aim of tackling maximum emergencies. Emergency services need to be also geared to handle vehicular accidents and cardiac-related emergencies, with both speed of service and quality of medical care being essential to save lives.
